# Sign‐tracking modulates reward‐related neural activation to reward cues, but not reward feedback

**DOI:** 10.1111/ejn.15787

**Published:** 2022-08-15

**Authors:** Jay J. Duckworth, Hazel Wright, Paul Christiansen, Abigail K. Rose, Nicholas Fallon

**Affiliations:** ^1^ Department of Psychology University of Liverpool Liverpool UK; ^2^ School of Psychology Liverpool John Moores University Liverpool UK

**Keywords:** fMRI, incentive salience, reward cues, selective attention, VMAC

## Abstract

Research shows cognitive and neurobiological overlap between sign‐tracking [value‐modulated attentional capture (VMAC) by response‐irrelevant, discrete cues] and maladaptive behaviour (e.g. substance abuse). We investigated the neural correlates of sign‐tracking in 20 adults using an additional singleton task (AST) and functional magnetic resonance imaging (fMRI). Participants responded to a target to win monetary reward, the amount of which was signalled by singleton type (reward cue: high value vs. low value). Singleton responses resulted in monetary deductions. Sign‐tracking—greater distraction by high‐value vs. low‐value singletons (*H > L*)—was observed, with high‐value singletons producing slower responses to the target than low‐value singletons. Controlling for age and sex, analyses revealed no differential brain activity across *H > L* singletons. Including sign‐tracking as a regressor of interest revealed increased activity (*H > L singletons*) in cortico‐subcortical loops, regions associated with Pavlovian conditioning, reward processing, attention shifts and relative value coding. Further analyses investigated responses to *reward feedback* (*H > L*). Controlling for age and sex, increased activity (*H > L reward feedback*) was found in regions associated with reward anticipation, attentional control, success monitoring and emotion regulation. Including sign‐tracking as a regressor of interest revealed increased activity in the temporal pole, a region related to value discrimination. Results suggest sign‐tracking is associated with activation of the ‘attention and salience network’ in response to reward cues but not reward feedback, suggesting parcellation between the two at the level of the brain. Results add to the literature showing considerable overlap in neural systems implicated in reward processing, learning, habit formation, emotion regulation and substance craving.

## Graphical abstract

Using an additional singleton task and fMRI, participants responded to targets to win monetary rewards, the amount of which was signalled by singleton type (high value vs. low value). Singleton responses resulted in monetary deductions. Sign‐tracking—greater distraction by high‐value vs. low‐value singletons (*H > L*)—was observed. Sign‐tracking correlated with increased BOLD response differentials across H > L singleton displays in regions associated with Pavlovian conditioning, reward processing, attention shifts and relative value coding. No such associations were found in response to reward feedback displays.

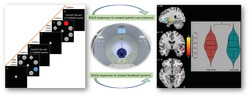

**Abbreviations**
AST: additional singleton task
AUD: alcohol use disorder
fMRI: functional magnetic resonance imaging
GT: goal‐tracking
OFC: orbitofrontal cortex
RT: response/reaction time
ST: sign‐tracking
SUD: substance use disorder
VMAC: value‐modulated attentional capture

## 1 INTRODUCTION

The human attentional system is capable of processing chaotic displays and can locate a target cue (or cue feature) while ignoring all other distracting information in a fraction of a second (Pessoa, 2014; Theeuwes, 2010). Cues—specifically discrete, response‐irrelevant, but reward‐associated cues—can also become imbued with value, which in turn amplifies cue salience leading to interference (via distraction) of goal‐oriented visual search, a finding that has been linked to reward‐based disorders such as substance dependence (Anderson et al., 2013; Le Pelley et al., 2016). This co‐opting of attentional resources by discrete reward‐paired cues (or *signals*), sometimes even when the actual reward itself is present, is termed sign‐tracking. The use of such cues merely to predict the onset of reward is termed goal‐tracking. In humans, sign‐tracking procedures typically measure reflexive, unconscious, bottom‐up attentional allocation to cues (Albertella et al., 2017; Le Pelley et al., 2015; Pearson et al., 2015), rather than the active, conscious, bodily approach measured in non‐human animal paradigms (Boakes et al., 1978; Breland & Breland, 1961; Brown & Jenkins, 1968; Williams & Williams, 1969), although see Moher et al. (2016). Here, we investigate how the human brain operates when engaged in sign‐tracking and explore how this may shed light on the underlying components of reward‐related disorder.

Proclivity for sign‐tracking has been identified in those with disordered eating, risky substance use, depression and HIV+ diagnosis, highlighting potentially important links between sign‐tracking and maladaptive behaviours (Albertella et al., 2017, 2019; Anderson et al., 2013; Anderson, Kronemer, et al., 2016; Anderson, Leal, et al., 2014; Versace et al., 2015). Investigating the neurobiological basis of these phenotypes (animals, at least, tend to reliably cluster into observable sign‐tracking and goal‐tracking groups), a recent review of animal research concluded that sign‐tracking is dependent on dopamine and subcortical circuits (bottom‐up, in the cognitive sense), and goal‐tracking more on cortical circuits (top‐down) (Flagel & Robinson, 2017). Although this may explain why sign‐tracking is associated with maladaptive behaviours such as risky alcohol and substance use, work on the neurophysiology of sign‐tracking in humans is in its infancy.

Most functional magnetic resonance imaging (fMRI) studies employing variations of the additional singleton task (AST; the primary measure of sign‐tracking in humans) investigate questions related to, but not identical to, sign‐tracking (Anderson, 2016; de Fockert et al., 2004; de Fockert & Theeuwes, 2012). Anderson (2016) used only a ‘training phase’ version of the AST wherein participants were explicitly instructed to respond *to* the singletons, whereas de Fockert et al. (2004) and de Fockert and Theeuwes (2012) did not pair singletons to reward; rather, colour singletons were distracting merely due to their visual distinctiveness. To our knowledge, in the only fMRI investigation to directly explore sign‐tracking, two AST experiments first trained participants to attend to target colours (one high value, one low value), before using these target colours as singleton distractors in a subsequent test phase (Anderson, Laurent, & Yantis, 2014). During Experiment 1, participants received feedback during the training phase, and sign‐tracking was related to activation in the intraparietal sulcus, extrastriate visual cortex, caudate tail and primary motor cortex. Experiment 2 was almost identical, except no reward feedback during training. Experiment 2 found no sign‐tracking effect nor any associated neural activations, suggesting Experiment 1 results were due to the direct influence of reward value and not merely stimulus–response habits carried over from training to test phase (i.e. selection and reward history effects), which frequently muddies interpretation (Kim & Anderson, 2019; Theeuwes, 2019). Replicating these fMRI results, a study using positron emission tomography (PET) found that value‐modulated attentional capture (VMAC) by such cues was associated with activity in the dorsal striatum (Anderson, Kuwabara, et al., 2016). Importantly, the aforementioned reward regions are also heavily implicated in substance‐related states and behaviours, such as craving and drug seeking (Volkow et al., 2006; Wong et al., 2006).

The current study builds on this previous neuroimaging work by improving and extending several parameters. Firstly, a modified AST presents reward‐paired singletons (high vs. low value) that are never rewarded as targets and that, if responded to, result in active punishment, thus preventing selection/reward history effects and superstitious conditioning (see Section 2). Secondly, a larger sample size and identical number of trials ensured improved statistical power. Finally, investigation into how VMAC is represented at the level of the brain is captured via two novel analyses. First, direct contrast analyses assessed neural responses towards high‐value vs. low‐value singletons possessing reward values >0, contrasting Anderson, Laurent, and Yantis (2014) who compared value (>0) with no value (0). Second, neural responses to both reward‐associated singletons (conditioned stimuli) and reward feedback are considered, the latter previously only conducted using PET (Anderson, Kuwabara, et al., 2016). Although comparison of neural activations towards reward‐associated cues and reward feedback is commonplace in fMRI using such tasks as monetary incentive delay (Kahnt et al., 2010; Knutson et al., 2001, 2003), no study to date has investigated the comparison using the modified AST. The current study will provide a breakdown of how incentive salience—the attribution of desire to, or “wanting” of, a stimulus—is represented at the level of the brain across different reward displays.

We predicted greater blood‐oxygenated‐level‐dependent (BOLD) signal contrast for high‐value compared with low‐value singleton trials (time‐locked to stimulus onset) in the dorsal striatum and the early visual and frontal cortices, as found by previous AST studies (Anderson, 2016; Anderson, Kuwabara, et al., 2016). Analyses time‐locked to reward feedback were exploratory, although given the aforementioned PET analysis (Anderson, Kuwabara, et al., 2016) we predicted greater BOLD response in reward‐related circuitry (specifically in the dorsal striatum) on high‐value vs. low‐value feedback trials, which would indicate a symmetry in neural response to displays of direct reward (reward feedback) and indirect reward (reward‐associated cues).

## 2 MATERIALS AND METHODS

### 2.1 Participants

Twenty participants (10 female) aged 19–34 (*M* = 25.85 ± 4.58) were recruited from a previous, virtually identical, larger (*N* = 114) AST study (Duckworth et al., *in preparation*). Response time (RT) data from this previous study were analysed by way of a tertiary split to allow us to recruit participants to the current study whose task performance was suboptimal (sign‐trackers), moderate (intermediates) and optimal (goal‐trackers), allowing for variation in responses. Specifically, the split was applied to the ‘RT Bias’ outcome variable (*RT Bias = target response times on high‐value trials − target response times on low‐value trials*), our primary outcome measure in both the previous and current study. An opportunity sample of participants from each of the three categories was then recruited to the present study (time between studies approximately 2 years). Inclusion criteria were normal/corrected‐to‐normal vision and right‐handedness (self‐reported). Exclusion criteria were self‐reported past or present alcohol‐ or drug‐use problems, brain surgery, crushing head injury, pregnancy, diagnosis of a neurological disease, psychiatric illness, or anxiety disorder (e.g. claustrophobia, PTSD, etc.) and surgery, meaning they could not be exposed to the scanner's magnetic field (e.g. pacemaker, metal implants, immovable body piercings, etc.). Participants provided informed consent prior to participation, approved by the University of Liverpool Research Ethics Committee.

### 2.2 AST (see Figure 1)

**FIGURE 1:**
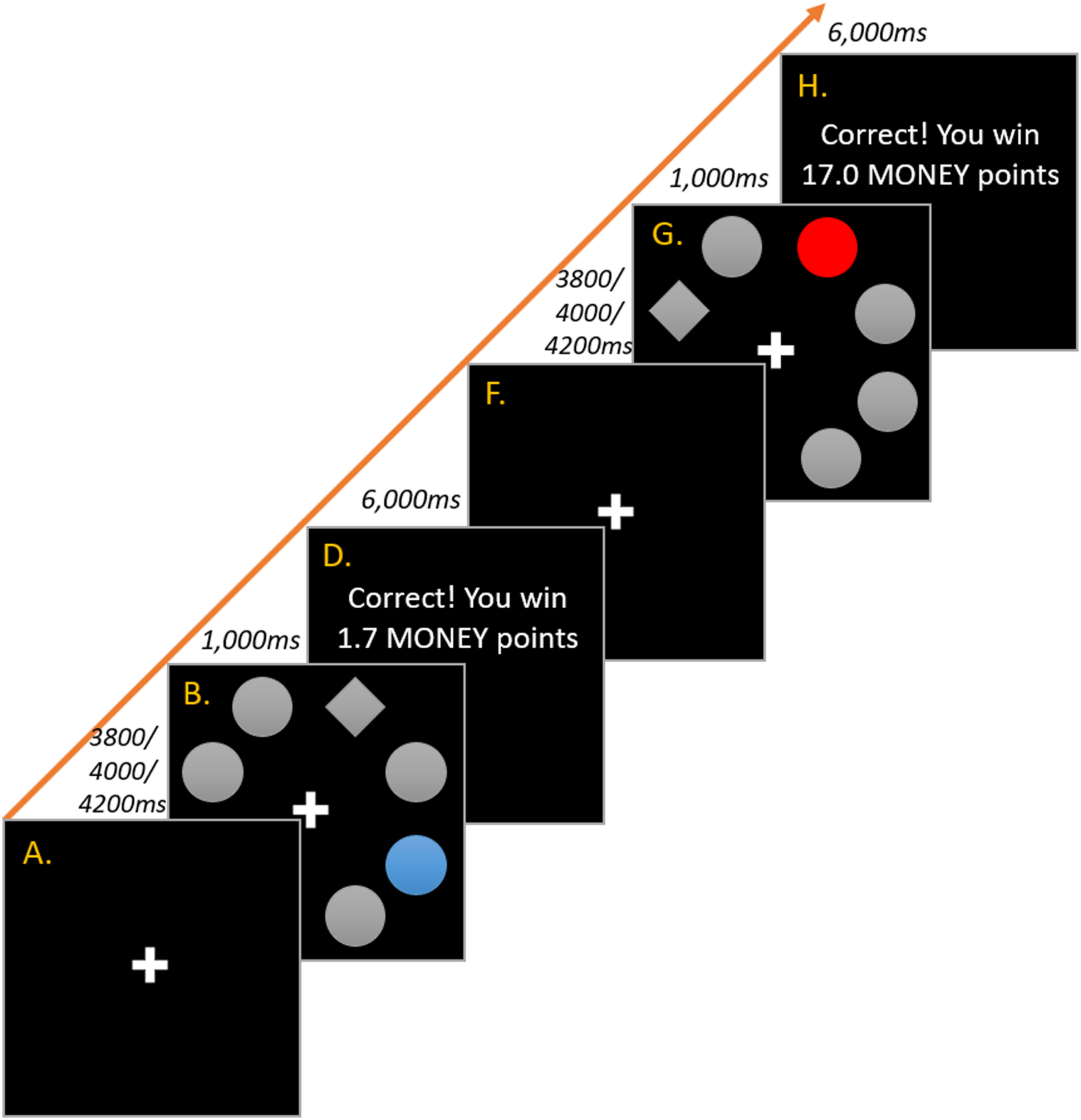
Two AST trials: here, blue trial = low value; red trial = high value. (a) Fixation (4000 ms, randomly staggered +/−200 ms). (b) Stimulus‐showing a blue singleton (target/diamond response = top‐right button; omission/singleton response = bottom‐right). (c) Post‐trial interval (*not shown;* 2000 ms). (d) Feedback showing (correct) low‐value feedback. (e) Pre‐trial interval (*not shown;* 2000 ms). (f) Fixation. (g) Stimulus‐onset showing a red singleton (target/diamond response = top left; omission/singleton response = top‐right). (h) Feedback showing (correct) high‐value feedback. Feedback values show reward as if reaction times were identical across trials.

#### 2.2.1 Task run‐through

Task run‐through was coded in Inquisit v.3.0.6.0 (Millisecond Software, 2011). Eight stimuli are presented on each trial: one grey diamond (shape singleton; target), one coloured circle [colour singleton (red/blue); conditioned stimulus] and six grey circles (valueless distractors). Participants were instructed to find the diamond target and avoid the circles and informed that the faster they found the target the more points they won, with 10× more points available on high‐value trials (e.g. red singleton) compared with low‐value trials (e.g. blue singleton). Colour singleton responses resulted in loss of points (5 points deducted across both trial types); responses corresponding to neutral distractors resulted in a timeout and loss of 5 points if participants did not then immediately key press the correct response within the time limit. The task was presented on a projector screen parallel to the top of the participant's head (~60 cm away), which they viewed in a mirror attached to the head coil. The task was presented in landscape at 1024 × 768 resolution. In addition to six practice trials, there were 160 true trials (80 high value, 80 low value) in a fixed sequence, the order of which was determined by a random sequence generator. The design is similar to previous AST fMRI studies (Anderson, 2016; de Fockert et al., 2004). Participants responded via a four‐button response box (held in their right hand) organised in a square pattern, with each button corresponding to a quadrant of the screen. Task duration ~41 min.

#### 2.2.2 Comparison with other ASTs

This AST variant most closely resembles an amalgamation of several versions used by Le Pelley and colleagues (Albertella et al., 2017, 2019; Le Pelley et al., 2015; Pearson et al., 2015). Although the features listed here are not themselves unique, their collation into a single task for use with fMRI is, to our knowledge, novel. This AST has no training phase; coloured stimuli (singletons) are *always* response irrelevant. Note, however, that although participants had completed this task previously (see Section 2.1), this does not count as a ‘training phase’ as participants were never trained to attend to a colour (i.e. red/blue were never the target), but rather were actively deterred from doing so across both studies. Value‐colour contingencies were kept consistent for each participant across studies, given evidence of persistent effects (Anderson & Yantis, 2013). That reward‐paired cues are never actively targeted prevents *selection history* effects, and the fact that responses to colours are never rewarded prevents *reward history* effects. Our AST uses continuous—as opposed to categorical—reward feedback based on RT to the target, preventing ‘covert distractor shifts’ and superstitious conditioning (Le Pelley et al., 2015; Skinner, 1948). Finally, our AST actively punishes singleton responses (i.e. pressing a key that corresponds to the location of a singleton), meaning that any sign‐tracking is observed *despite* active punishment.

### 2.3 Procedure

Before attending the laboratory, participants gave consent to complete online questionnaires via Qualtrics, Provo, UT, USA (2015) to determine inclusion/exclusion criteria. Testing took place between 9:00 and 18:00 in LiMRIC (Liverpool Magnetic Resonance Imaging Centre) at the University of Liverpool. All participants gave written consent for a second time and underwent safety screening by a senior radiographer. During the lab session, participants completed the AST while undergoing functional scanning. The Single Ease Question (SEQ) was completed after the AST, asking: ‘Overall, how easy or difficult did you find this task? Circle a number below’, with a 7‐point Likert scale from 1 to 7 (*very easy* to *very difficult*) (Sauro & Dumas, 2009). Upon completion, participants received £50 compensation. Sessions lasted ~60 min.

### 2.4 Data acquisition, reduction and analysis

#### 2.4.1 AST

Measures were those used by Le Pelley et al. (2015). Outcomes were divided into ‘Omissions Bias’ and ‘RT Bias’. Omissions Bias is the ratio of direct singleton responses (omissions) participants made towards high‐value vs. low‐value singletons (*frequency of high‐value singleton omissions − frequency of low‐value singleton omissions*), whereas RT Bias is RT towards the diamond target across singleton trial types (*target RTs on high‐value trials − target RTs on low‐value trials*). All errors due to either lack of responding or responding to neutral distractors until trial timeout were excluded. Greater (positive) values indicate sign‐tracking (as higher‐value singleton distractors should induce higher singleton engagement and slower target responses than lower‐value distractors). RTs < 150 ms were recoded as missing data to ensure exclusion of anticipatory responses (Le Pelley et al., 2015; Roper et al., 2014). Given stimuli were presented for 1000 ms, no upper cut‐off was applied.

### 2.5 Imaging parameters: Data acquisition, pre‐processing and analysis

#### 2.5.1 Acquisition

Magnetic resonance images were acquired using a whole‐body 3‐T Siemens Trio MRI imaging system (Siemens, Magnetom, Erlangen, Germany) and an eight‐channel head coil. A localiser scan followed by clinical T1‐ and T2‐weighted anatomical scans were acquired (total acquisition time: 14:42 min). A high‐resolution three‐dimensional T1‐weighted image was acquired using a modified driven equilibrium Fourier transform (MDEFT) sequence [time to repeat (TR) = 7.92 ms; time to echo (TE) = 2.48 ms; flip angle = 16°; 176 sagittal slices; slice thickness = 1 mm; matrix 256 × 240 × 176; in‐plane voxel size 1 × 1 × 1 mm; field of view (FoV) = 256 mm]. T1 scans were used for co‐registering BOLD images and were also evaluated by a qualified clinician for medical anomalies or incidental findings that would require further investigation in line with LiMRIC research governance and protocols. Finally, fMRI scanning was conducted while participants completed the AST, with the task projected ~60 cm away (37 axial slices; 7.4 mm spacing; field of view = 192 mm; TR = 2200 ms; TE = 30 ms; voxel size = 3 × 3 × 3 mm; FoV = 192 mm; volumes per run = 1,20; total acquisition time: 41 min).

#### 2.5.2 Pre‐processing

Spatial pre‐processing of data was performed in SPM12 (Statistical Parametric Mapping, Welcome Trust Centre for Neuroimaging, University College London, UK) running in MATLAB R2020a (MathWorks Inc., Natick, USA, 2020). Functional volumes underwent slice‐timing correction, realignment and unwarping, normalisation to MNI (Montreal Neurological Institute) space using the normalised EPI (echo planar imaging) co‐registered to each participant's T1 image and spatial smoothing (5‐mm full‐width at half‐maximum Gaussian kernel filter). The SPM default temporal high‐pass filter (>128 s) was applied to the time series to remove slow signal drifts (confounds). Locations of brain activations in MNI space are described according to the Harvard–Oxford Cortical and Subcortical Atlases (Desikan et al., 2006; Frazier et al., 2005; Goldstein et al., 2007; Makris et al., 2006). Multi‐image Analysis Graphical User Interface (MANGO, v.4.1) was used create final images.

#### 2.5.3 Analysis

##### First‐level analysis (within‐subject)

Analyses were conducted in SPM12. An event‐related design was employed. In the *Stimulus‐Onset Analysis*, high‐value and low‐value trials served as regressors of interest in a fixed‐effects general linear model. Similarly, in the *Feedback‐Onset Analysis*, onset times for high‐value and low‐value feedback were input into the model. The six motion parameters derived from the realignment pre‐processing step were included as covariates to account for motion‐related variance; these were evaluated, and a motion artefact threshold (translation >3 mm, rotation >1 degree) was employed for exclusion of data with excessive movement. No participants met criteria for exclusion. Regressors were convolved with a hemodynamic response function and subjected to a first‐level analysis using a fixed‐effects general linear model (GLM), generating within‐subject contrasts via a paired‐samples *t*‐test for the contrast *high value > low value*.

##### Second‐level analysis (between‐subject)

Factorial design specifications were created for *Stimulus‐Onset* and *Feedback‐Onset* analyses. A univariate *t*‐test determined group‐level differences between high‐value and low‐value trials using individual contrast maps. Inputting the within‐subject contrasts of the first‐level into the second‐level factorial design accounts for both within‐ and between‐subject variability, and thus our approach overall was one of a random‐effects GLM (Penny et al., 2007). This two‐level approach is commonly used, including by the authors of the software (SPM) used here (Chumbley et al., 2014; Henson & Penny, 2005). The factorial design specifications were implemented twice for each analysis. Model 1 included age and sex as *regressors of no interest*, a common practice in fMRI research due to evidence of the effects (even when small) of these factors on intrinsic network activity, functional connectivity and moderation of outcomes (Dubois et al., 2018; Filippi et al., 2013; Zhang et al., 2016). This allows for a straightforward *high‐value > low‐value* comparison while controlling for obvious demographic factors. Model 2 again controlled for age and sex, but further included RT Bias (sign‐tracking) as a *regressor of interest* wherein sign‐tracking is correlated with participants' beta values from the high‐value > low‐value contrast. This whole‐brain correlation analysis allows investigation into how brain activations differ across the high‐value > low‐value contrast *as a function of sign‐tracking*. Whole‐brain analyses employ *α* < 0.001 (uncorrected), with extent thresholds of *k* ≥ 10, and surviving clusters were confirmed using small‐volume cluster‐level analysis with familywise error (FWE) correction [volume of interest (VOI) sphere, 5 mm radius] at *α* < 0.05 (Anderson, 2016; Anderson, Laurent, & Yantis, 2014; de Fockert et al., 2004; Woo et al., 2014).

## 3 RESULTS

### 3.1 Behavioural data

**FIGURE 2:**
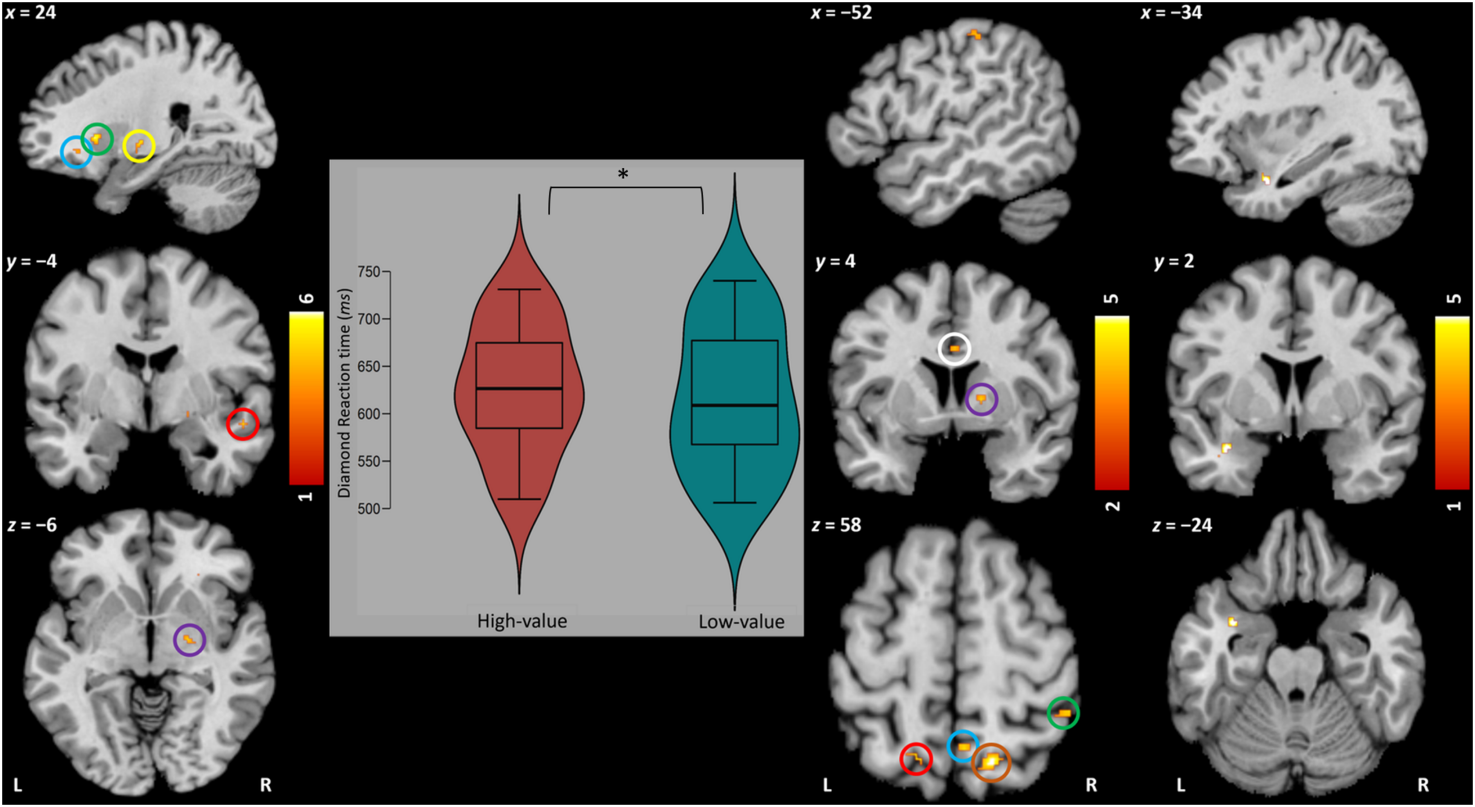
Behavioural and BOLD analyses. Violin plot shows RT to target/diamond on high‐ vs. low‐value trials (*p < 0.05). **24, −4, −6 (Stimulus‐Onset, Model 2):** *blue*: R orbitofrontal cortex; *green*: R putamen; *yellow*: R pallidum; *red*: R anterior/posterior superior temporal gyri; *purple*: R amygdala. **−52, 4, 58 (Feedback‐Onset, Model 1):** *Top*: L postcentral gyrus /anterior supramarginal gyrus; *white*: R anterior cingulate gyrus; *purple*: R pallidum; *red*: L lateral superior occipital cortex/superior parietal lobule; *blue:* R precuneus cortex; *orange*: R lateral superior occipital cortex; *green*: R posterior supramarginal gyrus/angular gyrus. **−34, 2, −24 (Feedback‐Onset, Model 2):** All coordinates: L temporal pole. Colour bar = *t*‐value gradient; all clusters confirmed via small‐volume cluster‐level analysis with FWE‐correction (VOI sphere, 5 mm radius), *p* < 0.05.

Analyses conducted in SPSS v.24.0 (IBM Corp., 2017) and JASP v.0.9.2.0 (JASP Team, 2020) investigated whether RTs were slower on high‐ vs. low‐value trials (Figure 2). A paired‐samples *t*‐test showed that target RTs were slower on high‐value vs. low‐value trials (*M* = 625.17 ± 68.01 vs. *M* = 615.78 ± 76.47), *t*(19) = 1.96, *p* = 0.03, *d*_*z*_ = 0.44 (1‐tailed). Bayesian analysis (using a *t*‐distribution Oosterwijk prior centred at 0.350, scale = 0.102 and *df* = 3, given previous effect size estimations as small‐to‐moderate) revealed ‘moderate’ evidence in favour of the sign‐tracking hypothesis (BF_+0_ = 4.51, 95% credible interval: 0.142, 0.670). Frequentist and Bayesian statistics provide evidence that attention was co‐opted to a greater extent by discrete, response‐irrelevant high‐value compared with low‐value singletons (i.e. sign‐tracking). Analyses concerning omissions could not be performed due to insufficient cases; across all participants and all trials, there were only 12 high‐value omissions and one low‐value omission, amounting to 0.4% of all responses. A relatively lengthy trial duration of 1 s possibly resulted in low task difficulty and thus few omissions, which is supported by 80% of participants rating the task ≤3 out of seven on the SEQ difficulty scale. Omissions are not considered in future analyses.

Given that participants were recruited and classified as sign‐trackers (*n* = 6), goal‐trackers (*n* = 10) or intermediates (*n* = 4) based on previous AST performance (Duckworth et al., *in preparation*), we determined the test–retest reliability of RT Bias scores across the current and previous 1 studies. An intraclass correlation (ICC) estimate was attained using an average‐measures (*k* = 2), consistency‐type, two‐way mixed‐effects model, ICC = 0.50, *p* = 0.07, 95% CI [−0.26, 0.80]. Results indicate poor‐to‐moderate reliability (Koo & Li, 2016). In addition to the inferential statistics, it is worth noting that only 30% of goal‐trackers remained goal‐trackers across time, compared with 50% of sign‐trackers and intermediates, suggesting tracking behaviour is either unstable, has not been faithfully measured across time or both.

### 3.2 Imaging data

#### 3.2.1 Stimulus‐onset analysis

Brain activity was time‐locked to stimulus‐onset (diamond target|colour singleton|neutral distractors; Figure 1, Events B/G), with singleton type (high‐value vs. low‐value) the key difference across trials. In Model 1, a whole‐brain analysis contrasted *high‐value > low‐value* trials, controlling for the influence of age and sex. At a cluster‐forming height threshold of *T* = 3.65 (*p* < 0.001 uncorrected, *k* ≥ 10 voxels), no suprathreshold clusters were observed. Results indicate no observed difference in neural activity in response to the presentation of singletons signalling different levels of monetary reward (Table 1).

**TABLE 1:** fMRI analyses assessing BOLD response to high‐value vs. low‐value cues and feedback

Region^a^	MNI coordinates			k	*T*‐value
	x	y	z		
**Stimulus‐onset analysis**					
*M1: Main effect of condition (high > low): Adjusted for age and sex* ^b^					
No suprathreshold clusters					
*M2: Correlation between RT Bias and T‐value (high > low): Adjusted for age and sex* ^c^					
R putamen	24	18	0	27	5.89
R pallidum	26	−14	−2	21	5.75
*Peak*: R amygdala	22	−4	−10		4.07
R anterior/posterior superior temporal gyri	52	−4	−14	12	4.90
R orbitofrontal cortex	28	30	−8	15	4.36
*Peak*: R orbitofrontal cortex	24	28	−10		4.26
**Feedback‐onset analysis**					
*M1: Main effect of condition (high > low): Adjusted for age and sex* ^d^					
R anterior cingulate gyrus	2	0	28	46	6.04
R lateral superior occipital cortex	14	−62	60	41	5.53
	36	−66	46	29	4.74
*Peak*: R lateral superior occipital cortex	32	−62	54		4.42
L lateral superior occipital cortex|superior parietal lobule	−16	−60	58	11	4.50
R pallidum	16	4	2	10	4.47
R posterior supramarginal gyrus|angular gyrus	48	−42	58	10	4.46
L postcentral gyrus|anterior supramarginal gyrus	−52	−28	50	13	4.16
R precuneus cortex	4	−58	58	16	4.61
*M2: Correlation between RT Bias and T‐value (high > low): Adjusted for age and sex* ^e^					
L cerebral cortex|temporal pole	−34	2	−24	22	5.78

*Notes*: Positive *T*‐values indicate high > low, whereas negative values indicate low > high. Activations in white matter structures are not reported.

^a^ Neuromorphometrics reported using Harvard–Oxford Cortical and Subcortical Atlas; only structures with probabilities ≥10% reported; L/R (left/right hemispheres). k = cluster size (voxels), minimum extent threshold ≥10; voxel size: 2 mm^3^; M1/M2 = Models 1/2; age and sex entered as regressors of no interest.

^b^ Height threshold: T = 3.65 (*p* < 0.001, uncorrected).

^c^ Height threshold: T = 3.69 (*p* < 0.001, uncorrected).

^d^ Height threshold T = 3.65 (*p* < 0.001 uncorrected).

^e^ Height threshold: T = 3.69 (*p* < 0.001, uncorrected); all clusters were confirmed using small‐volume cluster‐level analysis with FWE correction (VOI sphere, 5 mm radius), *p* < 0.05.

Model 2 additionally included RT Bias as a regressor of interest to assess responses to high‐value vs. low‐value cues as a function of sign‐tracking. At a height threshold of *T* = 3.69 (*p* < 0.001 uncorrected, *k* ≥ 10 voxels), four clusters were observed (all *high>low*): right putamen (*Cluster 1*), right pallidum with peak activation in the right amygdala (*Cluster 2*), right anterior and posterior superior temporal gyri (*Cluster 3*) and the right orbitofrontal cortex (*Cluster 4*). Clusters were confirmed using small‐volume cluster‐level analysis with FWE‐correction, *p* < 0.05 (Table 1 and Figure 2). Results indicate that differential neural activity to high‐ vs. low‐value reward cues is moderated by one's susceptibility to VMAC/sign‐tracking.

#### 3.2.2 Feedback‐onset analysis

A second series of analyses considered brain activity time‐locked to reward feedback (Figure 1, Events D/H). Model 1 contrasted activation differences towards high‐value relative to low‐value reward feedback, controlling for age and sex. At a height threshold of *T* = 3.65 (*p* < 0.001 uncorrected, *k* ≥ 10), seven clusters were found (all *high>low*): right anterior cingulate gyrus (*Cluster 1*), right lateral superior occipital cortex (*Cluster 2*), left lateral superior occipital cortex/ left superior parietal lobule (*cluster 3*), right pallidum (*Cluster 4*), right posterior supramarginal gyrus /right angular gyrus (*Cluster 5*), left postcentral gyrus/left anterior supramarginal gyrus (*Cluster 6*) and the right precuneus cortex (*Cluster 7*). Clusters were confirmed using small‐volume cluster‐level analysis with FWE‐correction, *p* < 0.05 (Table 1 and Figure 2). Results indicate widespread differentiation in neural response to high‐value vs. low‐value feedback.

Model 2 added RT Bias as a regressor of interest. At a height threshold of *T* = 3.69 (*p* < 0.001 uncorrected, *k* ≥ 10 voxels), one cluster was observed (*high>low*): left cerebral cortex/temporal pole. Clusters were confirmed using small‐volume cluster‐level analysis with FWE‐correction, *p* < 0.05 (Table 1 and Figure 2). Results indicate that activity in left temporal pole alone was linked to tendency to sign‐track.

## 4 DISCUSSION

Using an event‐related fMRI design, we investigated the neural correlates of VMAC towards discrete, reward‐associated, response‐irrelevant cues (i.e. sign‐tracking). Results revealed a small‐to‐moderate reaction time sign‐tracking effect, with slower target responses on trials containing irrelevant high‐value, relative to low‐value, singletons (reward cues). Whole‐brain analyses investigated neural responses during two phases of the AST: (i) stimulus‐onset: presentation of reward‐associated cues and (ii) feedback‐onset: presentation of monetary reward. Stimulus‐onset analyses initially revealed no statistically significant activation differences in response to high‐ vs. low‐value singletons; however, when assessing neural activation across high‐ vs. low‐value trials *as a function of participants' sign‐tracking*, regions involved in motivation, reward processing, relative value encoding and attention shifting were revealed (Bechara et al., 2002; Farrell et al., 2019; Lim et al., 2013; Perry et al., 2014; Rapuano et al., 2016; Smith et al., 2009; Tindell et al., 2006). Feedback‐onset analyses showed somewhat opposing results: Initial analyses showed differential activation across high‐ vs. low‐value reward feedback in regions associated with reward anticipation, attentional control, monitoring of success and emotion regulation (Anderson, 2016; Caminiti et al., 1996; Cao et al., 2018; Sahan et al., 2019; Seghier, 2013; Travers et al., 2015). However, when viewing neural responses as a function of participants' sign‐tracking, only activation differences in the temporal pole were found, a region involved in distinguishing between levels of reward.

### 4.1 Neural correlates of VMAC by high‐value vs. low‐value singletons (sign‐tracking)

The stimulus‐onset analysis controlling for age and sex (Model 1) showed no statistically significant activation differences across trial types, suggesting similar responses to singletons of different value (Table 1). However, when participants' sign‐tracking (i.e. distractibility by high‐value > low‐value singletons) was added as a regressor of interest (Model 2), four clusters were more active in response to high‐ vs. low‐value reward cues. This suggests sign‐tracking is associated with a neural signature, with certain regions of activation specific to the behaviour and not just a response to mere cue presentation. Increased activation was observed in the right hemispheric putamen, pallidum (peaking in the amygdala), anterior and posterior superior temporal gyri and orbitofrontal cortex (OFC) (Table 1).

The putamen (a substructure of the dorsolateral striatum) is associated with implicit and reinforcement learning, behavioural preferences for certain foods, differential responding to high‐ vs. low‐value reward presentation and the processing of reward‐associated distractors (Anderson, 2015; Mattfeld et al., 2011; O'Doherty et al., 2006; Pollmann et al., 2016). The pallidum plays a role in reward processing, encoding hedonic reward, incentive salience, and the striato‐pallidal complex (containing substructures like the putamen and pallidum) reflects incentive (motivation) levels and is described as a major reward‐related network (Richard et al., 2016; Schmidt et al., 2012; Smith et al., 2009; Tindell et al., 2006). The right amygdala is involved in the anticipation of responding for a reward (vs. no reward), evaluating reward outcome, and the amygdala–ventral striatum complex plays a vital role in stimulus–reward (incentive) learning (Bechara et al., 2002; Cador et al., 1989; Knutson et al., 2001, 2003; Liu et al., 2011). Furthermore, preclinical work shows greater neuronal activity (via *c‐fos* expression) in the basolateral amygdala of sign‐trackers relative to goal‐trackers in response to reward cues (Flagel & Robinson, 2017). The right anterior and posterior superior temporal gyri are associated with attention, salience detection, activity in response to reward cues, shifting attention in tasks that involve encoding relative value and processing semantic (but not aesthetic) information (Krebs et al., 2011; Lim et al., 2013; Rapuano et al., 2016). Finally, the OFC encodes the current value of reward‐associated cues and contributes to decision making, and the dorsolateral striatum (wherein the putamen resides) mediates OFC activity resulting in shifts from goal‐directed to habitual behaviour (Gottfried et al., 2003; Gremel & Costa, 2013; O'Doherty, 2007).

Importantly, many of these regions have also been implicated in reward‐related maladaptive behaviours and outcomes, such as substance abuse and use disorders (SUDs). For example, dopamine activity in the putamen is associated with cue‐elicited drug craving *and* predicts magnitude of sign‐tracking (Anderson, Kuwabara, et al., 2016; Volkow et al., 2006; Wong et al., 2006). The right ventral pallidum and putamen are associated with reward‐seeking behaviours for alcohol and drugs and relapse to cocaine in rats (Farrell et al., 2019; Perry et al., 2014; Smith et al., 2009). The dorsolateral striatum is involved in the observable shift in individuals' substance‐related behaviour from goal‐directed to habitual (Corbit & Janak, 2016), whereas the putamen differs in size between adolescents diagnosed with alcohol use disorder (AUD) and non‐drinking controls (Fein et al., 2013). Similarly, the superior temporal gyrus shows reduced volume in adolescents with AUD compared with controls (Brooks & Stein, 2016). Finally, the amygdala is involved in cue‐elicited substance craving (Bonson et al., 2002; Grant et al., 1996), whereas the OFC is implicated in the progression and maintenance of SUDs (Volkow & Fowler, 2000). Such results suggest neurobiological overlap between sign‐tracking and components (e.g. craving) of reward‐related disorder (Albertella et al., 2017, 2019).

### 4.2 Neural responses to high‐value vs. low‐value reward feedback

Controlling for age and sex (Model 1), seven clusters were found to be more active in response to high‐ vs. low‐value feedback. Increased activation was observed in the right‐hemispheric anterior cingulate gyrus, pallidum, posterior supramarginal gyrus/angular gyrus and precuneus cortex and the left‐hemispheric postcentral gyrus/anterior supramarginal gyrus, superior parietal lobule and the bilateral superior occipital cortex. Including sign‐tracking as a regressor of interest (Model 2), only left‐hemispheric cerebral cortex/temporal pole activation was seen, suggesting differential responding in this region is dependent on individuals' tendency to sign‐track.

As detailed previously, the pallidum is associated with reward processing and encoding incentive salience (Richard et al., 2016; Smith et al., 2009). Several of the gyri (right posterior supramarginal gyrus/angular gyrus and left postcentral gyrus/anterior supramarginal gyrus) are related to attention, emotion regulation, abstract representations of emotion, number processing and reading, consistent with participants interpreting a multilevel (*H > L*) reward feedback display (Cao et al., 2018; Henderson et al., 2015; Seghier, 2013). The right anterior cingulate gyrus plays a role in reward anticipation and attention allocation to both emotional and purely attentional stimuli and as an interface between motor control, drive and cognition (Paus, 2001; Yamasaki et al., 2002). The left superior parietal lobule influences visual processing, learning, orienting spatial attention and working memory representation during number comparison (Caminiti et al., 1996; Sahan et al., 2019; Travers et al., 2015). The lateral superior occipital cortex (right side in particular) is crucial in value‐modulated attentional priority, and previous studies show increased activation for similar *high‐value > low‐value* reward contrasts employed here (Anderson, 2015; Pollmann et al., 2016). The precuneus cortex is associated with reward expectation during an AST and is involved in visuospatial integration as a functional core of the ‘Default Mode Network’ (Anderson, 2016; Bruner et al., 2017; Li et al., 2019). Finally, the left temporal pole is implicated in the modulation of affect and the maintenance of semantic memory and is posited to act as a bridge between sensory perceptions and emotional content in memory (Olson et al., 2007). It also regulates salience in the Default Mode Network alongside structures like the precuneus (Geng et al., 2017). Collectively, these results suggest that participants were absorbing and encoding the relative value information contained in the reward feedback display and potentially preparing the attentional system for similar displays in the future, priming top‐down control. Crucially, sign‐tracking behaviour may be directly influenced by the temporal pole's regulation of salience.

These regions are also implicated in reward‐based disorders. Greater activation of the bilateral superior occipital cortex in response to high‐ vs. low‐value feedback is supported by virtually identical results across various demographics (Smoski et al., 2009). The postcentral gyrus shows differential activation across a range of reward presentations (from food to various drugs) and demographics (obese vs. healthy weight; substance dependent vs. healthy control) (García‐García et al., 2014), whereas both the right angular gyrus and left superior parietal lobule contain greater neurite density in binge drinkers vs. healthy controls (Morris et al., 2018). Cocaine abusers undergoing withdrawal show reduced activity in the anterior cingulate gyrus (Volkow & Fowler, 2000) and activation in the anterior cingulate and precuneus in response to high‐ vs. low‐value reward predicted relapse in methamphetamine‐dependent patients (Gowin et al., 2015). Finally, supramarginal gyrus (general) activity is related to regular use of stimulant drugs (Ersche et al., 2020), and the temporal pole differs in size between cocaine users and controls (Geng et al., 2017).

### 4.3 Task specificity and sign‐tracking

A small‐to‐moderate RT sign‐tracking effect was observed, similar to previous results (Anderson, Laurent, & Yantis, 2014). However, most existing neuroimaging work employing ASTs were not designed to measure sign‐tracking directly (i.e. automatic attentional capture by discrete, reward‐associated, response‐irrelevant cues). For example, previous studies presented singletons not associated with value (de Fockert et al., 2004; de Fockert & Theeuwes, 2012; Lavie & de Fockert, 2006), singletons presented as targets rather than distractors (Anderson, 2016), did not control for confounds such as selection and reward history effects, or collapsed high‐ and low‐value trials (Anderson, Laurent, & Yantis, 2014; Kim & Anderson, 2019). Uniquely, we measured neural responses to singletons of high‐value and low‐value, giving real‐time continuous reward feedback based on accuracy and speed, and actively punished responses to reward‐associated cues (i.e. sign‐tracking).

### 4.4 Limitations and future directions

Small sample size limits statistical power, potentially inflating the false discovery rate, deflating true positive discovery and/or producing imprecise effect size estimates. This problem is pervasive in fMRI primarily due to high operational costs (Button et al., 2013). Further, we used a button‐box version of the AST, but our work has shown eye‐tracking is more internally reliable (Christiansen et al., 2015). Future work should use eye‐tracking measures in larger samples.

## 5 CONCLUSION

This study employed an AST to investigate the neural correlates of sign‐tracking using fMRI. The presentation of high‐value vs. low‐value discrete, response‐irrelevant, reward‐associated cues (singletons) elicited activation in regions related to motivation (incentive salience), reward and visual selective attention. Activation in these regions was correlated with participants' tendency to sign‐track (i.e. become distracted by high‐value vs. low‐value cues). Novel analyses revealed neural activation in response to high‐ vs. low‐value reward feedback in regions linked to number comparison, encoding hedonic value and value‐modulated attentional priority, although most were not directly associated with participants' sign‐tracking. Both displays (reward‐linked *cues* and reward *feedback*) evoked activation in regions also found in substance users in response to substance‐related cues and that are also associated with factors underlying consumption (e.g. craving). This study makes a unique contribution to the field and suggests that one's propensity to sign‐track is related to one's neural response to reward‐linked cues, but not necessarily to reward feedback. The regions identified may help elucidate the role of habituated cognitive processes in reward‐related disorders at the level of the brain.

## CONFLICTS OF INTEREST

The authors have no competing interests to declare.

## AUTHOR CONTRIBUTIONS

JJD, AR, NF, PC and HW were responsible for the study's conception and design. JJD collected the data and conducted analysis of behavioural data. JJD, NF and HW conducted the functional imaging analysis. JJD and NF produced the fMRI figures. JJD, NF, PC and AR provided interpretations of results. JJD drafted the manuscript. All authors provided critical revision of the manuscript for important intellectual content. All authors critically reviewed content and approved the final version for publication.

### PEER REVIEW

The peer review history for this article is available at https://publons.com/publon/10.1111/ejn.15787.

## Data Availability

All data can be accessed via Open Science Framework through the following link: https://osf.io/am5gr/?view_only=256994d43fb0431f9fb01aed46265a2e.

## Footnotes

^1^
In the previous study, participants completed an almost identical AST (sans extended trial durations, inter‐trial intervals and extra valueless distractors), except that they made responses solely via eye movements. This reliability analysis is the only use of these previous (Duckworth et al., in preparation) data in the current study.
